# Correlates of verbal and physical violence experienced and perpetrated among cisgender college women: serial cross-sections during one year of the COVID-19 pandemic

**DOI:** 10.3389/frph.2024.1366262

**Published:** 2024-07-25

**Authors:** Deborah A. Theodore, Craig J. Heck, Simian Huang, Yuije Huang, April Autry, Brit Sovic, Cynthia Yang, Sarah Ann Anderson-Burnett, Caroline Ray, Eloise Austin, Joshua Rotbert, Jason Zucker, Marina Catallozzi, Delivette Castor, Magdalena E. Sobieszczyk

**Affiliations:** ^1^ Division of Infectious Diseases, Department of Medicine, Columbia University Irving Medical Center, New York, NY, United States; ^2^ Department of Epidemiology, Columbia University Mailman School of Public Health, New York, NY, United States; ^3^ Barnard College, Health & Wellness, Barnard College, New York, NY, United States; ^4^ Division of Child and Adolescent Health, Department of Pediatrics, Columbia University Irving Medical Center, New York, NY, United States; ^5^ Heilbrunn Department of Population & Family Health, Columbia University Mailman School of Public Health, New York, NY, United States

**Keywords:** adolescent girls and young women, longitudinal analysis, college health, emotional violence, racial disparity

## Abstract

**Introduction:**

Violence against women is a prevalent, preventable public health crisis. COVID-19 stressors and pandemic countermeasures may have exacerbated violence against women. Cisgender college women are particularly vulnerable to violence. Thus, we examined the prevalence and correlates of verbal/physical violence experienced and perpetrated among cisgender women enrolled at a New York City college over one year during the COVID-19 pandemic.

**Methods:**

From a prospective cohort study, we analyzed data self-reported quarterly (T1, T2, T3, T4) between December 2020 and December 2021. Using generalized estimated equations (GEE) and logistic regression, we identified correlates of experienced and perpetrated violence among respondents who were partnered or cohabitating longitudinally and at each quarter, respectively. Multivariable models included all variables with unadjusted parameters *X*^2^ *p*-value ≤0.05.

**Results:**

The prevalence of experienced violence was 52% (T1: *N* = 513), 30% (T2: *N* = 305), 33% (T3: *N* = 238), and 17% (T4: *N* = 180); prevalence of perpetrated violence was 38%, 17%, 21%, and 9%. Baseline correlates of experienced violence averaged over time (GEE) included race, living situation, loneliness, and condom use; correlates of perpetrated violence were school year, living situation, and perceived social support. Quarter-specific associations corroborated population averages: living with family members and low social support were associated with experienced violence at all timepoints except T4. Low social support was associated with higher odds of perpetrated violence at T1/T3. Other/Multiracial identity was associated with higher odds of violence experience at T3.

**Conclusions:**

Living situation was associated with experienced and perpetrated violence in all analyses, necessitating further exploration of household conditions, family dynamics, and interpersonal factors. The protective association of social support with experienced and perpetrated violence also warrants investigation into forms of social engagement and cohesion. Racial differences in violence also require examination. Our findings can inform university policy development on violence and future violence research. Within or beyond epidemic conditions, universities should assess and strengthen violence prevention and support systems for young women by developing programming to promote social cohesion.

## Introduction

Violence against women and girls is a pervasive, preventable public health problem. Global and United States national data show that one in three women have survived physical, sexual, or psychological intimate partner violence (IPV) or non-partner sexual violence in their lifetime (1, 2). Rising levels of violence against women were reported following the onset of the COVID-19 pandemic (3, 4). According to one systematic review and meta-analysis, lockdown policies were followed by an 8.1% increase in domestic violence (5). Consequences of the pandemic, such as stay-at-home orders, school closures, social isolation, financial insecurity, and substance use in the context of increased stress and/or mental illness, may have contributed to increased rates of violence. The impact of COVID-19 on violence may be attenuated by the socioeconomic vulnerability of families and women from minoritized communities (4, 6–8). Individuals diagnosed with COVID-19 were more likely to experience violence, possibly related to other social determinants of both SARS-CoV-2 acquisition and the epidemic of interpersonal violence (9, 10). Similarly, increases in IPV have been observed in previous infectious disease outbreaks and crisis situations (e.g., during outbreaks of Ebola, Cholera, Zika, and Nipah virus, and in the setting of earthquakes and other natural disasters) (9–12).

College-aged women faced relatively high levels of psychological distress and are vulnerable to intimate and non-intimate partner violence (13, 14). In Fall 2019, a national cross-sectional survey of American college students revealed that 12% of females experienced verbal violence from partners and 9% from non-partners, whereas 3% and 2% survived physical violence from these respective perpetrators (15). These statistics have remained relatively stable across independent samples throughout COVID-19 (16–18). Notably, most available data on violence survivorship among college women during the COVID-19 pandemic are cross-sectional, representing a single time point during the pandemic; few longitudinal studies have been published (10, 19).

Violence perpetrated (i.e., committed/enacted) by women is a less researched phenomenon. Historical data suggest between 10%–40% of college women perpetrate physical IPV, while between 40%–90% enact emotional violence (20). Although its measurement is negatively affected by stereotypes surrounding femininity, gender, and heteronormativity, evidence implies that woman-perpetrated sexual violence might not be a rare occurrence (21, 22). Research also supports that adverse childhood events and maladaptive personality traits and attitudes facilitate woman-perpetrated sexual violence (23–25).

Little is known about the longitudinal patterns and determinants of experienced and enacted violence among college women during COVID-19 pandemic. Measures of socializing (e.g., participation in organized sports, relationship status, perceived social support) have been associated with elevated vulnerability to violence. While the relationship between violence and certain health-risk behaviors is well established, associations with other aspects of health behaviors, including self-efficacy (e.g., condom use, hormonal contraceptive use), remain unclear temporally and in the pandemic context (26–28). The debate continues about the co-incident or causal relationship between pandemic behavior modification (e.g., substance use, changes in sexual behavior as a result of social distancing recommendations) and violence experience or perpetration (29–31). Additionally, the immediate and long-term consequences of violence, particularly at the formative stage of late adolescence and young adulthood, need to be elucidated.

Thus, we estimated the prevalence and social, psychological, and behavioral correlates of experienced and perpetrated physical and verbal violence among college women at successive stages of the COVID-19 pandemic. We hypothesized that during the COVID-19 pandemic, experienced and perpetrated violence would be high, associated with social connectivity, stability and health behaviors, and these associations would be modified over time.

## Methods

### Study design

From December 2020 to December 2021, this longitudinal cohort study prospectively followed college students, faculty, and staff affiliated with a New York City (NYC) residential college. Study methods have been previously described (13). In brief, emails containing study details and enrollment links were distributed in December 2020-January 2021 (T1) to everyone with an active institutional email address. Eligible participants were (1) enrolled students or employed faculty or staff, (2) at least 18 years old, (3) able to speak and understand English, and (4) able to provide written informed consent. After T1, participants were surveyed quarterly: March-April 2021 (T2), July-August 2021 (T3), and November-December 2021 (T4). This article adheres to the reporting standards established within The Strengthening the Reporting of Observational Studies in Epidemiology (STROBE) Statement (Supplementary Table S1) (32).

### Participants

We restricted the analytic sample to students who self-identified as cisgender women and those either in relationships or living with family, friends, roommates, suitemates, or significant others (cohabitating) when the survey was administered.

### Data collection

All data were collected by an anonymous, self-administered questionnaire that included sociodemographic, physical status, social, and psychological well-being information. All data were collected and managed using Research Electronic Data Capture (REDCap) (33).

### Outcome measures

We explored two outcome measures: experienced violence and perpetrated violence. Topics surrounding violence were explored using questions developed around quarantine in another COVID-19 study and other emergency contexts, such as Hurricane Katrina (34, 35). Violence-related outcomes were asked only of respondents who were cohabiting or in a relationship.

We operationalized experienced violence, the primary outcome, as a partner, spouse, or cohabitating person subjecting the respondent to physical or verbal violence in the past 30 days. Physical violence included being pushed, grabbed, hit, slapped, kicked, or having something thrown at the respondent. Verbal violence involved yelling or saying things that make the other person feel bad, embarrassed, or frightened. The initial Likert response of, “Very Often, “Fairly Often, Sometimes, Almost Never, Never” was dichotomized into ever vs. never experienced violence in the past 30 days. We defined perpetrated violence, the co-primary outcome, as the respondent enacting physical or verbal violence on a partner, spouse, or cohabitating person in the past 30 days as defined above. Similarly, we dichotomized responses as ever vs. never perpetrated violence in the last 30 days. In keeping with other published analyses of violence, we further examined the joint occurrence of experiencing and perpetuating violence (36).

### Correlates

Detailed descriptions of all correlates are presented in Supplementary Table S2. Demographic variables included age, ethnicity, race, school year at enrollment, living situation, and financial aid status. Social variables were relationship status, social group involvement, sports group involvement, loneliness, and social support. Substance use variables were tobacco smoking or vaping, alcohol consumption, and frequency of any of the following drugs used in the past 30 days: marijuana, cocaine, painkillers, heroin, sedatives, stimulants, club drugs, hallucinogens, or inhalants. Sexual behavior variables were recent sexual activity, condom use, and change in sexual behavior due to COVID-19. We also examined self-care/care-seeking behaviors based on COVID-19 symptoms and hormonal contraception use. The sociodemographic correlates phrasing and response items were aligned with NIH reporting guidelines. Sexual and drug use behavior correlates were drawn from standardized questions from the National Institute on Drug Abuse (NIDA). At the time of questionnaire development, there were limitations regarding availability of scales that were applicable and/or validated for use in the pandemic context.

### Statistical methods

We described the sample using proportions for categorical variables and medians and interquartile ranges (IQR) for continuous variables.

Because pandemic countermeasures, mortality/morbidity rates, and interventions varied greatly over the cohort's observation period, we performed serial cross-sectional analyses of each quarterly survey rather than analyzing repeated measures. We conducted unadjusted and adjusted logistic regression analyses to estimate correlates of experienced and perpetrated violence, including correlates with a *X*^2^ *p*-values ≤0.05 for parameters in the adjusted model.

To estimate the average change in violence outcomes over time, we conducted a generalized estimating equations models (GEE) with an autoregressive correlation matrix and using the baseline values of the independent variables, carried forward. Only those with non-missing outcome data across all timepoints were included in the GEE models.

We performed all data management, transformation, and analysis in R (v.4.3.2).

## Results

### Sample characteristics

Of the 666 respondents that completed the T1 survey, 513 comprised the analytic sample after removing respondents who were faculty [*n* = 35], staff [*n* = 69)]), identified as transgender (*n* = 22), not living with others and not in a relationship (*n* = 22), or missing outcome data (*n* = 5). For the GEE analyses, 120 students had complete outcome data across quarters.

Table 1 shows the characteristics of the study sample at T1. The median age of participants was 20 (IQR: 19–21). Participants were predominantly White-identifying (62%) non-Hispanic (86%) students not on financial aid (57%), with even distribution across school years. Majority were single (70.6%) and living with others (58% with family, 40% with peers). Social group involvement was high (62%), while sports involvement was rare (14%). Loneliness (62%) and social support networks (74%) were common, whereas smoking (7%), alcohol consumption (31%), drug use (20%), hormonal contraception use (36%), and recent sexual activity (38%) were rare. Among those recently sexually active (*n* = 193), approximately half used condoms (52%, 101/193), and most reported that COVID-19 affected their sexual activities in some way (99%, 191/193). Of those who had ever experienced COVID-19 symptoms (*n* = 111), approximately half sought care from medical professionals (52%) and about one-third isolated but did not seek care (32%).

**Table 1:** Sample characteristics and experience and perpetration of violence at T1 (December 2020–January 2021).

Characteristic	Overall, *N* = 513	Experienced violence					Perpetrated violence				
		Did not experience, *N* = 247	Experienced, *N* = 266	OR (95% CI)	*p*-value	aOR (95% CI)	Did not perpetrate, *N* = 317	Perpetrated, *N* = 196	OR (95% CI)	*p*-value	aOR (95% CI)
Age	20.00 (19.00, 21.00)	20.00 (19.00, 21.00)	20.00 (18.00, 21.00)	0.9 (0.78, 1.03)	0.13		20.00 (19.00, 21.00)	20.00 (19.00, 21.00)	0.99 (0.86, 1.14)	>0.9	
Race											
White	309 (62%)	166 (69%)	143 (56%)	1.00 (ref)		1.00 (ref)	202 (65%)	107 (57%)	1.00 (ref)		
Asian	104 (21%)	41 (17%)	63 (25%)	**1.78** (**1.14, 2.82)**	**0**.**012**	1.25 (0.77, 2.06)	57 (18%)	47 (25%)	1.56 (0.99, 2.45)	0.055	
Other/multiracial	84 (17%)	33 (14%)	51 (20%)	**1.79** (**1.10, 2.95)**	**0**.**02**	1.51 (0.90, 2.56)	50 (16%)	34 (18%)	1.28 (0.78, 2.10)	0.3	
Ethnicity											
Not Hispanic	434 (86%)	214 (87%)	220 (85%)	1.00 (ref)			275 (88%)	159 (83%)	1.00 (ref)		
Hispanic	72 (14%)	33 (13%)	39 (15%)	1.15 (0.70, 1.90)	0.6		39 (12%)	33 (17%)	1.46 (0.88, 2.42)	0.14	
School year											
Senior	125 (24%)	68 (28%)	57 (21%)	1.00 (ref)			78 (25%)	47 (24%)	1.00 (ref)		
First-year	139 (27%)	61 (25%)	78 (29%)	1.53 (0.94, 2.49)	0.089		83 (26%)	56 (29%)	1.12 (0.68, 1.84)	0.7	
Sophomore	113 (22%)	52 (21%)	61 (23%)	1.4 (0.84, 2.34)	0.2		67 (21%)	46 (23%)	1.14 (0.68, 1.92)	0.6	
Junior	136 (27%)	66 (27%)	70 (26%)	1.27 (0.78, 2.06)	0.3		89 (28%)	47 (24%)	0.88 (0.53, 1.45)	0.6	
Living situation											
With peers/significant other	206 (40%)	129 (52%)	77 (29%)	1.00 (ref)		1.00 (ref)	152 (48%)	54 (28%)	1.00 (ref)		1.00 (ref)
With family	295 (58%)	108 (44%)	187 (71%)	**2.90** (**2.01, 4.21)**	**<0**.**001**	**2.50** (**1.68, 3.73)**	155 (49%)	140 (72%)	**2.54** (**1.74, 3.76)**	**<0**.**001**	**2.44** (**1.59, 3.80)**
Alone	10 (2.0%)	9 (3.7%)	1 (0.4%)				9 (2.8%)	1 (0.5%)			
Financial aid	215 (43%)	93 (39%)	122 (47%)	1.39 (0.97, 1.99)	0.071		127 (42%)	88 (45%)	1.13 (0.79, 1.63)	0.5	
Current Relationship status											
In a relationship	151 (29%)	71 (29%)	80 (30%)	1.00 (ref)			91 (29%)	60 (31%)	1.00 (ref)		
Not in a relationship	362 (71%)	176 (71%)	186 (70%)	0.94 (0.64, 1.37)	0.7		226 (71%)	136 (69%)	0.91 (0.62, 1.35)	0.6	
Social group involvement											
None	194 (38%)	89 (36%)	105 (39%)	1.00 (ref)			124 (39%)	70 (36%)	1.00 (ref)		
At least one social group	319 (62%)	158 (64%)	161 (61%)	0.86 (0.60, 1.23)	0.4		193 (61%)	126 (64%)	1.16 (0.80, 1.68)	0.4	
Sports involvement											
None	403 (79%)	188 (76%)	215 (81%)	1.00 (ref)			240 (76%)	163 (83%)	1.00 (ref)		
At least one sports group	71 (14%)	34 (14%)	37 (14%)	0.95 (0.57, 1.58)	0.8		48 (15%)	23 (12%)	0.71 (0.41, 1.19)	0.2	
Missing	39 (7.6%)	25 (10%)	14 (5.3%)				29 (9.1%)	10 (5.1%)			
Since 3 months ago, feel…											
Less lonely	195 (38%)	106 (43%)	89 (34%)	1.00 (ref)		1.00 (ref)	128 (40%)	67 (35%)	1.00 (ref)		
Same	105 (21%)	50 (20%)	55 (21%)	1.31 (0.81, 2.11)	0.3	1.04 (0.62, 1.75)	65 (21%)	40 (21%)	1.18 (0.72, 1.92)	0.5	
Lonelier	211 (41%)	91 (37%)	120 (45%)	**1.57** (**1.06, 2.33)**	**0**.**024**	1.15 (0.74, 1.78)	124 (39%)	87 (45%)	1.34 (0.90, 2.01)	0.2	
Has a social support network											
Yes	376 (74%)	196 (81%)	180 (68%)	1.00 (ref)		1.00 (ref)	243 (78%)	133 (68%)	1.00 (ref)		1.00 (ref)
Uncertain	64 (13%)	27 (11%)	37 (14%)	1.49 (0.88, 2.57)	0.14	1.39 (0.77, 2.53)	38 (12%)	26 (13%)	1.25 (0.72, 2.14)	0.4	1.17 (0.64, 2.10)
No	66 (13%)	20 (8.2%)	46 (17%)	**2.5** (**1.45, 4.48)**	**0**.**001**	**2.53** (**1.38, 4.83)**	30 (9.6%)	36 (18%)	**2.19** (**1.29, 3.74)**	**0**.**004**	**2.31** (**1.34, 4.14)**
Current smoking	35 (6.8%)	20 (8.1%)	15 (5.6%)	0.68 (0.33, 1.35)	0.3		22 (7.0%)	13 (6.6%)	0.95 (0.46, 1.91)	0.9	
Alcohol consumption											
Never/rare	353 (69%)	162 (66%)	191 (72%)	1.00 (ref)			215 (68%)	138 (70%)	1.00 (ref)		
Moderate/high	160 (31%)	85 (34%)	75 (28%)	0.75 (0.51, 1.09)	0.13		102 (32%)	58 (30%)	0.89 (0.60, 1.30)	0.5	
Drug use											
Never/rare	409 (80%)	191 (77%)	218 (82%)	1.00 (ref)			248 (78%)	161 (82%)	1.00 (ref)		
Monthly/weekly/daily	104 (20%)	56 (23%)	48 (18%)	0.75 (0.51, 1.09)	0.13		69 (22%)	35 (18%)	0.78 (0.49, 1.22)	0.3	
Recent sexual activity											
No	310 (61%)	144 (58%)	166 (63%)	1.00 (ref)			181 (57%)	129 (66%)	1.00 (ref)		
Yes	193 (38%)	100 (40%)	93 (35%)	0.81 (0.56, 1.16)	0.2		132 (42%)	61 (31%)	**0.65** (**0.44, 0.94)**	**0**.**025**	
Missing	9 (1.8%)	3 (1.2%)	6 (2.3%)				4 (1.3%)	5 (2.6%)			
Condom use status											
Not sexually active	319 (64%)	146 (61%)	173 (67%)	1.00 (ref)			186 (60%)	133 (70%)	1.00 (ref)		1.00 (ref)
Sexually active, not using condoms	80 (16%)	43 (18%)	37 (14%)	0.73 (0.44, 1.19)	0.2		57 (18%)	23 (12%)	**0.56** (**0.33, 0.95)**	**0**.**035**	0.6 (0.32, 1.09)
Sexually active, using condoms	101 (20%)	51 (21%)	50 (19%)	0.83 (0.53, 1.30)	0.4		67 (22%)	34 (18%)	0.71 (0.44, 1.13)	0.2	0.95 (0.55, 1.62)
Response to COVID-19 symptoms											
Did not seek healthcare or isolate	17 (3.3%)	5 (2.0%)	12 (4.5%)	1.00 (ref)			7 (2.2%)	10 (5.1%)	1.00 (ref)		1.00 (ref)
Never had symptoms	402 (78%)	210 (85%)	192 (72%)	0.38 (0.12, 1.05)	0.075		263 (83%)	139 (71%)	**0.37** (**0.13, 0.98)**	**0**.**048**	0.36 (0.12, 1.00)
Isolated	36 (7.0%)	14 (5.7%)	22 (8.3%)	0.65 (0.18, 2.19)	0.5		20 (6.3%)	16 (8.2%)	0.56 (0.17, 1.78)	0.3	0.55 (0.15, 1.85)
Sought healthcare	58 (11%)	18 (7.3%)	40 (15%)	0.93 (0.26, 2.91)	0.9		27 (8.5%)	31 (16%)	0.8 (0.26, 2.38)	0.7	1.06 (0.32, 3.39)
Effects of COVID-19 on sexual behavior											
No effect	309 (61%)	143 (58%)	166 (64%)	1.00 (ref)			184 (59%)	125 (64%)	1.00 (ref)		
Any effect	191 (38%)	100 (40%)	91 (35%)	0.78 (0.55, 1.12)	0.2		128 (41%)	63 (32%)	0.72 (0.50, 1.05)	0.094	
Other	8 (1.6%)	4 (1.6%)	4 (1.5%)	0.86 (0.20, 3.70)	0.8		2 (0.6%)	6 (3.1%)	4.42 (1.00, 30.5)	0.072	
Current hormonal contraceptive use	183 (36%)	95 (39%)	88 (33%)	0.79 (0.55, 1.13)	0.2		117 (37%)	66 (34%)	0.86 (0.59, 1.25)	0.4	

Median (IQR); *n* (%).

For living situation, we omitted the alone response category from all regressions due to small cell sizes.

When recent sexual activity and condom use behaviors were both eligible for multivariable inclusion, we opted for condom use behaviors because it was more informative.

Bold values are statistically significant.

### Adjusted correlates of experienced and perpetrated violence

#### T1

**Figure 1:**
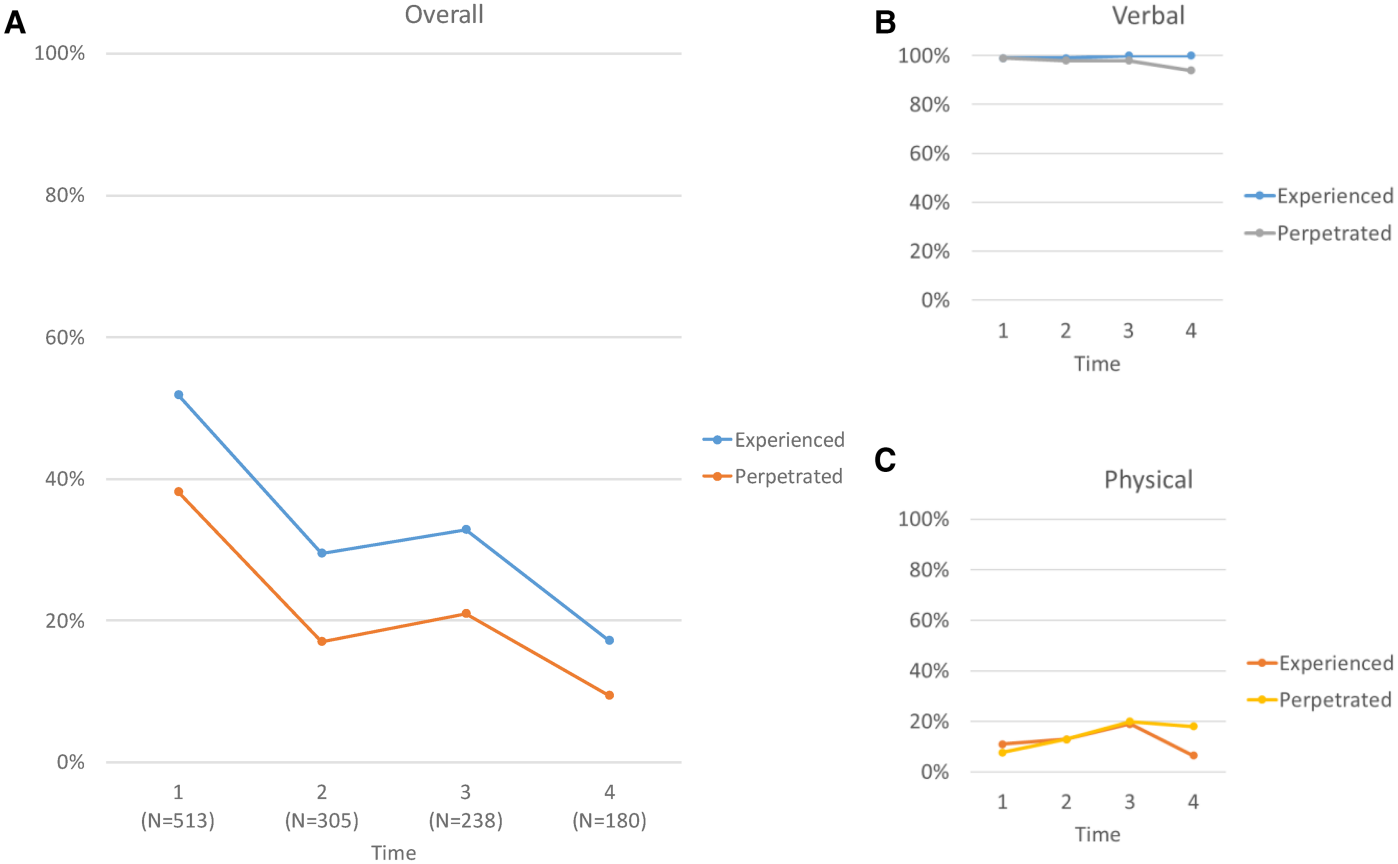
Experience and perpetration of violence over time. Panel **A** shows the joint experience of verbal and physical violence (blue) and the joint perpetration of verbal and physical violence (orange). Panel **B** shows the experience (blue) and perpetration (gray) of verbal violence. Panel **C** shows the experience (orange) and perpetration (yellow) of physical violence.

Table 1 also contains the descriptive, unadjusted, and adjusted analyses of experienced and perpetrated violence at T1. Overall, 52% (266/513) of respondents experienced violence [89% verbal, 10% poly-victimization (verbal & physical), 1% physical] (Figure 1). In the adjusted model, living with family vs. peers/significant others [aOR=2.50 (1.68–3.73)] and lack of social support [aOR = 2.53 (1.38–4.83)] were associated with experienced violence.

Comparatively, 38% (196/513) reported perpetrating violence (92% verbal, 7% poly-perpetration, 1% physical). After adjustment, living with family vs. peers/significant others [aOR = 2.44 (1.59, 3.80)] and lack of social support [aOR = 2.31 (1.34, 4.14)] were associated with increased perpetration.

#### T2

Table 2 highlights experienced and perpetrated violence during the second survey. Overall, 30% (90/305) reported experiencing violence (87% verbal, 12% poly-victimization, 1% physical). Respondents living with family vs. peers/significant others [aOR = 4.95 (2.74–9.10)] and those not feeling strong social support (uncertain support: aOR = 2.47 [1.21–5.01]; no support: aOR = 2.75 [1.20–6.28]) had a higher odds of experiencing violence.

**Table 2:** Sample characteristics and experience and perpetration of violence at T2 (March-April 2021).

Characteristic	Overall, *N* = 305	Experienced violence					Perpetrated violence				
		Did not experience, *N* = 215	Experienced, *N* = 90	OR (95% CI)	*p*-value	aOR (95% CI)	Did not experience, *N* = 253	Perpetrated, *N* = 52	OR (95% CI)	*p*-value	aOR (95% CI)
Age	20.00 (19.00, 21.00)	20.00 (19.00, 21.00)	19.50 (18.25, 20.75)	0.93 (0.77, 1.13)	0.5		20.00 (19.00, 21.00)	20.00 (19.00, 21.00)	1.13 (0.90, 1.43)	0.3	
Race											
White	184 (63%)	133 (64%)	51 (61%)	1.00 (ref)			162 (66%)	22 (47%)	1.00 (ref)		–
Asian	59 (20%)	39 (19%)	20 (24%)	1.34 (0.70, 2.49)	0.4		44 (18%)	15 (32%)	**2.51** (**1.19, 5.22)**	**0**.**014**	1.69 (0.74, 3.75)
Other/multiracial	50 (17%)	37 (18%)	13 (15%)	0.92 (0.44, 1.83)	0.8		40 (16%)	10 (21%)	1.84 (0.78, 4.11)	0.15	1.73 (0.71, 4.01)
Ethnicity											
Not Hispanic	257 (86%)	179 (84%)	78 (90%)	1.00 (ref)	–		216 (86%)	41 (84%)	1.00 (ref)	–	
Hispanic	42 (14%)	33 (16%)	9 (10%)	0.63 (0.27, 1.32)	0.2		34 (14%)	8 (16%)	1.24 (0.50, 2.76)	0.6	
School year											
Senior	65 (21%)	46 (21%)	19 (21%)	1.00 (ref)			49 (19%)	16 (31%)	1.00 (ref)		
First-year	89 (29%)	60 (28%)	29 (32%)	1.17 (0.59, 2.37)	0.7		75 (30%)	14 (27%)	0.57 (0.25, 1.28)	0.2	
Sophomore	61 (20%)	44 (21%)	17 (19%)	0.94 (0.43, 2.03)	0.9		51 (20%)	10 (19%)	0.6 (0.24, 1.43)	0.3	
Junior	89 (29%)	64 (30%)	25 (28%)	0.95 (0.47, 1.93)	0.9		77 (31%)	12 (23%)	0.48 (0.20, 1.09)	0.081	
Living situation											
With peers/significant other	233 (76%)	180 (84%)	53 (59%)	1.00 (ref)		1.00 (ref)	206 (81%)	27 (52%)	1.00 (ref)		1.00 (ref)
With family	64 (21%)	27 (13%)	37 (41%)	**4.65** (**2.61, 8.41)**	**<0**.**001**	**4.95 (2.74, 9.10)**	40 (16%)	24 (46%)	**4.58** (**2.40, 8.76)**	**<0**.**001**	**3.71 (1.81, 7.58)**
Alone	8 (2.6%)	8 (3.7%)	0 (0%)				7 (2.8%)	1 (1.9%)			
Financial aid	120 (41%)	86 (42%)	34 (39%)	0.87 (0.52, 1.45)	0.6		102 (42%)	18 (35%)	0.75 (0.39, 1.39)	0.4	
Current relationship Status											
In a relationship	93 (31%)	66 (31%)	27 (30%)	1.00 (ref)			76 (30%)	17 (33%)	1.00 (ref)		
Not in a relationship	211 (69%)	148 (69%)	63 (70%)	1.04 (0.61, 1.80)	0.9		176 (70%)	35 (67%)	0.89 (0.47, 1.72)	0.7	
Social group involvement											
None	156 (51%)	109 (51%)	47 (52%)	1.00 (ref)			132 (52%)	24 (46%)	1.00 (ref)		
At least one social group	149 (49%)	106 (49%)	43 (48%)	0.94 (0.57, 1.54)	0.8		121 (48%)	28 (54%)	1.27 (0.70, 2.33)	0.4	
Sports involvement											
None	258 (85%)	183 (85%)	75 (83%)	1.00 (ref)			212 (84%)	46 (88%)	1.00 (ref)		
At least one sports group	20 (6.6%)	12 (5.6%)	8 (8.9%)	1.63 (0.62, 4.09)	0.3		17 (6.7%)	3 (5.8%)	0.81 (0.18, 2.55)	0.7	
Missing	27 (8.9%)	20 (9.3%)	7 (7.8%)				24 (9.5%)	3 (5.8%)			
Since 3 months ago, feel…											
Less lonely	139 (46%)	103 (48%)	36 (40%)	1.00 (ref)			116 (46%)	23 (45%)	1.00 (ref)		
Same	68 (22%)	48 (22%)	20 (22%)	1.19 (0.62, 2.26)	0.6		53 (21%)	15 (29%)	1.43 (0.68, 2.93)	0.3	
Lonelier	97 (32%)	64 (30%)	33 (37%)	1.48 (0.84, 2.60)	0.2		84 (33%)	13 (25%)	0.78 (0.37, 1.61)	0.5	
Has a social support network											
Yes	227 (74%)	170 (79%)	57 (63%)	1.00 (ref)		1.00 (ref)	191 (75%)	36 (69%)	1.00 (ref)		
Uncertain	46 (15%)	27 (13%)	19 (21%)	**2.1** (**1.08, 4.04)**	**0**.**027**	**2.47 (1.21, 5.01)**	39 (15%)	7 (13%)	0.95 (0.37, 2.18)	>0.9	
No	32 (10%)	18 (8.4%)	14 (16%)	**2.32** (**1.07, 4.95)**	**0**.**03**	**2.75 (1.20, 6.28)**	23 (9.1%)	9 (17%)	2.08 (0.85, 4.73)	0.092	
Current smoking	22 (7.2%)	15 (7.0%)	7 (7.8%)	1.12 (0.42, 2.77)	0.8		19 (7.5%)	3 (5.8%)	0.75 (0.17, 2.32)	0.7	
Alcohol consumption											
Never/rare	197 (65%)	145 (67%)	52 (58%)	1.00 (ref)			164 (65%)	33 (65%)	1.00 (ref)		
Moderate/high	107 (35%)	70 (33%)	37 (42%)	1.47 (0.88, 2.45)	0.14		89 (35%)	18 (35%)	1.01 (0.53, 1.87)	>0.9	
Drug use											
Never/rare	235 (78%)	167 (78%)	68 (77%)	1.00 (ref)			197 (78%)	38 (75%)	1.00 (ref)		
Monthly/weekly/daily	68 (22%)	48 (22%)	20 (23%)	1.02 (0.56, 1.83)	>0.9		55 (22%)	13 (25%)	1.23 (0.59, 2.41)	0.6	
Recent sexual activity											
No	187 (62%)	129 (60%)	58 (64%)	1.00 (ref)			158 (63%)	29 (56%)	1.00 (ref)		
Yes	111 (37%)	81 (38%)	30 (33%)	0.82 (0.49, 1.38)	0.5		90 (36%)	21 (40%)	1.27 (0.68, 2.35)	0.4	
Missing	6 (2.0%)	4 (1.9%)	2 (2.2%)				4 (1.6%)	2 (3.8%)			
Condom use status											
Not sexually active	191 (65%)	131 (63%)	60 (68%)	1.00 (ref)			160 (65%)	31 (61%)	1.00 (ref)		
Sexually active, not using condoms	48 (16%)	37 (18%)	11 (12%)	0.65 (0.30, 1.32)	0.3		42 (17%)	6 (12%)	0.74 (0.26, 1.77)	0.5	
Sexually active, using condoms	57 (19%)	40 (19%)	17 (19%)	0.93 (0.48, 1.75)	0.8		43 (18%)	14 (27%)	1.68 (0.80, 3.39)	0.2	
Response to COVID-19 symptoms											
Did not seek healthcare or isolate	5 (1.7%)	4 (1.9%)	1 (1.1%)	1.00 (ref)			5 (2.0%)	0 (0%)	0.00 (0.00, 0.00)	>0.9	
Never had symptoms	269 (89%)	192 (90%)	77 (86%)	1.6 (0.23, 31.6)	0.7		222 (88%)	47 (90%)	1.00 (ref)		
Isolated	9 (3.0%)	4 (1.9%)	5 (5.6%)	5 (0.48, 121)	0.2		8 (3.2%)	1 (1.9%)	0.59 (0.03, 3.33)	0.6	
Sought healthcare	20 (6.6%)	13 (6.1%)	7 (7.8%)	2.15 (0.25, 46.6)	0.5		16 (6.4%)	4 (7.7%)	1.18 (0.33, 3.39)	0.8	
Effects of COVID-19 on sexual behavior											
No effect	184 (62%)	131 (62%)	53 (60%)	1.00 (ref)			155 (62%)	29 (57%)	1.00 (ref)		
Any effect	110 (37%)	76 (36%)	34 (39%)	1.11 (0.66, 1.85)	0.7		88 (35%)	22 (43%)	1.34 (0.72, 2.46)	0.4	
Other	5 (1.7%)	4 (1.9%)	1 (1.1%)	0.62 (0.03, 4.30)	0.7		5 (2.0%)	0 (0%)	0.00 (0.00, 0.00)	>0.9	
Current hormonal contraceptive use	118 (39%)	82 (38%)	36 (40%)	1.1 (0.66, 1.82)	0.7		100 (40%)	18 (35%)	0.8 (0.42, 1.49)	0.5	

Median (IQR); *n* (%).

For living situation, we omitted the alone response category from all regressions due to small cell sizes.

Bold values are statistically significant.

Approximately 17% of respondents reported perpetrating violence (87% verbal, 12% poly-perpetration, 2% physical). After statistical adjustment, living with family vs. peers/significant others was the only variable associated with perpetrating violence [aOR = 3.71 (1.81–7.58)].

#### T3

Table 3 presents experienced and perpetrated violence at T3. Approximately 1 in 3 respondents experienced violence (81% verbal, 19% poly-victimization). Other/Multiracial identity [aOR = 6.01 (2.31–16.40)], living with family vs. peers/significant others [aOR = 4.77 (2.24–10.9)], and uncertain [aOR = 3.39 (1.33, 8.87)] or no [aOR = 5.23 (1.53–18.8)] social support were associated with higher odds of experienced violence.

**Table 3:** Sample characteristics and experience and perpetration of violence at T3 (July–August 2021).

Characteristic	Overall, *N* = 238	Experienced violence					Perpetrated violence				
		Did not experience, *N* = 160	Experienced, *N* = 78	OR (95% CI)	*p*-value	aOR (95% CI)	Did not perpetrate, *N* = 188	Perpetrated, *N* = 50	OR (95% CI)	*p*-value	aOR (95% CI)
Age	20.00 (18.00, 20.00)	20.00 (19.00, 20.00)	19.00 (18.00, 20.00)	0.91 (0.73, 1.13)	0.4		20.00 (18.00, 20.00)	19.00 (18.25, 20.00)	0.98 (0.76, 1.26)	>0.9	
Race											
White	141 (61%)	106 (68%)	35 (47%)	1.00 (ref)		1.00 (ref)	117 (64%)	24 (50%)	1.00 (ref)		
Asian	57 (25%)	34 (22%)	23 (31%)	**2.05** (**1.06, 3.94)**	**0**.**031**	2.00 (0.90, 4.49)	43 (24%)	14 (29%)	1.59 (0.74, 3.32)	0.2	
Other/multiracial	32 (14%)	16 (10%)	16 (22%)	**3.03** (**1.37, 6.73)**	**0**.**006**	**6.01** (**2.31, 16.4)**	22 (12%)	10 (21%)	2.22 (0.90, 5.20)	0.072	
Ethnicity											
Not Hispanic	201 (87%)	134 (86%)	67 (88%)	1.00 (ref)	—		157 (85%)	44 (92%)	1.00 (ref)	—	
Hispanic	31 (13%)	22 (14%)	9 (12%)	0.82 (0.34, 1.82)	0.6		27 (15%)	4 (8.3%)	0.53 (0.15, 1.44)	0.3	
School year											
Senior	38 (16%)	24 (15%)	14 (18%)	1.00 (ref)			28 (15%)	10 (20%)	1.00 (ref)		
First-year	80 (34%)	49 (31%)	31 (40%)	1.08 (0.49, 2.45)	0.8		61 (32%)	19 (38%)	0.87 (0.36, 2.18)	0.8	
Sophomore	47 (20%)	29 (18%)	18 (23%)	1.06 (0.44, 2.60)	0.9		38 (20%)	9 (18%)	0.66 (0.23, 1.85)	0.4	
Junior	73 (31%)	58 (36%)	15 (19%)	0.44 (0.18, 1.06)	0.067		61 (32%)	12 (24%)	0.55 (0.21, 1.45)	0.2	
Living situation											
With peers/significant other	101 (44%)	84 (55%)	17 (22%)	1.00 (ref)		1.00 (ref)	93 (51%)	8 (17%)	1.00 (ref)		1.00 (ref)
With family	125 (54%)	67 (44%)	58 (75%)	**4.28** (**2.32, 8.21)**	**<0**.**001**	**4.77** (**2.24, 10.9)**	87 (47%)	38 (81%)	**5.08** (**2.35, 12.3)**	**<0**.**001**	**7.18 (2.99, 20.4)**
Alone	5 (2.2%)	3 (1.9%)	2 (2.6%)				4 (2.2%)	1 (2.1%)			
Financial aid	98 (43%)	60 (39%)	38 (49%)	1.49 (0.86, 2.60)	0.2		73 (41%)	25 (51%)	1.53 (0.81, 2.89)	0.2	
Current relationship status											
In a relationship	79 (33%)	57 (36%)	22 (28%)	1.00 (ref)			60 (32%)	19 (38%)	1.00 (ref)		
Not in a relationship	158 (67%)	102 (64%)	56 (72%)	1.42 (0.80, 2.60)	0.2		127 (68%)	31 (62%)	0.77 (0.41, 1.49)	0.4	
Social group involvement											
None	150 (63%)	101 (63%)	49 (63%)	1.00 (ref)			123 (65%)	27 (54%)	1.00 (ref)		
At least one social group	88 (37%)	59 (37%)	29 (37%)	1.01 (0.58, 1.77)	>0.9		65 (35%)	23 (46%)	1.61 (0.85, 3.03)	0.14	
Sports involvement											
None	209 (88%)	142 (89%)	67 (86%)	1.00 (ref)			163 (87%)	46 (92%)	1.00 (ref)		
At least one sports group	12 (5.0%)	8 (5.0%)	4 (5.1%)	1.06 (0.27, 3.49)	>0.9		11 (5.9%)	1 (2.0%)	0.32 (0.02, 1.72)	0.3	
Missing	17 (7.1%)	10 (6.2%)	7 (9.0%)				14 (7.4%)	3 (6.0%)			
Since 3 months ago, feel…											
Less lonely	135 (57%)	90 (56%)	45 (58%)	1.00 (ref)			106 (56%)	29 (58%)	1.00 (ref)		
Same	51 (21%)	39 (24%)	12 (15%)	0.62 (0.28, 1.26)	0.2		43 (23%)	8 (16%)	0.68 (0.27, 1.55)	0.4	
Lonelier	52 (22%)	31 (19%)	21 (27%)	1.35 (0.70, 2.61)	0.4		39 (21%)	13 (26%)	1.22 (0.56, 2.54)	0.6	
Has a social support network											
Yes	185 (79%)	131 (83%)	54 (70%)	1.00 (ref)		1.00 (ref)	154 (83%)	31 (62%)	1.00 (ref)		1.00 (ref)
Uncertain	27 (11%)	13 (8.2%)	14 (18%)	**2.61** (**1.15, 5.99)**	**0**.**022**	**3.39** (**1.33, 8.87)**	16 (8.6%)	11 (22%)	**3.42** (**1.42, 8.03)**	**0**.**005**	**3.32 (1.30, 8.40)**
No	23 (9.8%)	14 (8.9%)	9 (12%)	1.56 (0.62, 3.77)	0.3	**5.23** (**1.53, 18.8)**	15 (8.1%)	8 (16%)	**2.65** (**1.03, 6.79)**	**0**.**042**	**6.73 (2.00, 23.8)**
Current smoking	23 (9.8%)	15 (9.5%)	8 (10%)	1.11 (0.43, 2.67)	0.8		16 (8.6%)	7 (14%)	1.77 (0.65, 4.44)	0.2	
Alcohol consumption											
Never/rare	149 (63%)	98 (61%)	51 (65%)	1.00 (ref)			117 (62%)	32 (64%)	1.00 (ref)		
Moderate/high	89 (37%)	62 (39%)	27 (35%)	0.84 (0.47, 1.46)	0.5		71 (38%)	18 (36%)	0.93 (0.48, 1.76)	0.8	
Drug use											
Never/rare	191 (81%)	129 (81%)	62 (81%)	1.00 (ref)			151 (81%)	40 (80%)	1.00 (ref)		
Monthly/weekly/daily	46 (19%)	31 (19%)	15 (19%)	1.01 (0.50, 1.98)	>0.9		36 (19%)	10 (20%)	1.05 (0.46, 2.23)	>0.9	
Recent sexual activity											
No	127 (54%)	78 (49%)	49 (63%)	1.00 (ref)			99 (53%)	28 (56%)	1.00 (ref)		
Yes	105 (44%)	78 (49%)	27 (35%)	**0.55** (**0.31, 0.96)**	**0**.**039**		84 (45%)	21 (42%)	0.88 (0.46, 1.66)	0.7	
Missing	5 (2.1%)	3 (1.9%)	2 (2.6%)				4 (2.1%)	1 (2.0%)			
Condom use status											
Not sexually active	127 (56%)	78 (52%)	49 (65%)	1.00 (ref)		1.00 (ref)	99 (56%)	28 (57%)	1.00 (ref)		
Sexually active, not using condoms	35 (16%)	29 (19%)	6 (8.0%)	**0.33** (**0.12, 0.80)**	**0**.**022**	0.38 (0.10, 1.18)	28 (16%)	7 (14%)	0.88 (0.33, 2.15)	0.8	
Sexually active, using condoms	63 (28%)	43 (29%)	20 (27%)	0.74 (0.39, 1.39)	0.4	1.67 (0.71, 3.98)	49 (28%)	14 (29%)	1.01 (0.48, 2.06)	>0.9	
Response to COVID-19 symptoms											
Did not seek healthcare or isolate	11 (4.6%)	8 (5.0%)	3 (3.9%)	1.00 (ref)			8 (4.3%)	3 (6.0%)	1.00 (ref)		
Never had symptoms	213 (90%)	144 (90%)	69 (90%)	1.28 (0.36, 5.97)	0.7		171 (91%)	42 (84%)	0.65 (0.18, 3.09)	0.5	
Isolated	3 (1.3%)	2 (1.3%)	1 (1.3%)	1.33 (0.05, 20.2)	0.8		1 (0.5%)	2 (4.0%)	5.33 (0.38, 144)	0.2	
Sought healthcare	10 (4.2%)	6 (3.8%)	4 (5.2%)	1.78 (0.28, 12.2)	0.5		7 (3.7%)	3 (6.0%)	1.14 (0.16, 8.07)	0.9	
Effects of COVID-19 on sexual behavior											
No effect	175 (74%)	114 (72%)	61 (79%)	1.00 (ref)			138 (74%)	37 (76%)	1.00 (ref)		
Any effect	56 (24%)	41 (26%)	15 (19%)	0.68 (0.34, 1.31)	0.3		45 (24%)	11 (22%)	0.91 (0.41, 1.89)	0.8	
Other	4 (1.7%)	3 (1.9%)	1 (1.3%)	0.62 (0.03, 4.98)	0.7		3 (1.6%)	1 (2.0%)	1.24 (0.06, 10.0)	0.9	
Current hormonal contraceptive use	95 (40%)	64 (40%)	31 (40%)	0.99 (0.57, 1.72)	>0.9		72 (38%)	23 (46%)	1.37 (0.73, 2.57)	0.3	

Median (IQR); *n* (%).

For living situation, we omitted the alone response category from all regressions due to small cell sizes.

When recent sexual activity and condom use behaviors were both eligible for multivariable inclusion, we opted for condom use behaviors because it was more informative.

Around 1 in 5 students perpetrated physical violence (80% verbal, 18% poly-perpetration, 2% physical). Reports of perpetrated violence were associated with living with family vs. peers/significant others [aOR = 7.18 (2.99–20.4)] and lacking social support (uncertain support: aOR = 3.32 [1.30–8.40]; no support: aOR = 6.73 [2.00–23.8]).

#### T4

Table 4 shows descriptive, unadjusted and adjusted associations at T4. Overall, 17% reported experiencing violence (94% verbal, 6% poly-victimization) and 9% reported perpetrating violence (82% verbal, 12% poly-perpetration, 6% physical). Ultimately, no factors were associated with violence experience.

**Table 4:** Sample characteristics and experience and perpetration of violence at T4 (November-December 2021).

Characteristic	Overall, *N* = 180	Experienced violence					Perpetrated violence				
		Did not experience, *N* = 149	Experienced, *N* = 31	OR (95% CI)	*p*-value	aOR (95% CI)	Did not perpetrate, *N* = 163	Perpetrated, *N* = 17	OR (95% CI)	*p*-value	aOR (95% CI)
Age	19.50 (19.00, 20.00)	19.00 (19.00, 20.00)	20.00 (19.00, 20.50)	1.07 (0.78, 1.47)	0.7		19.00 (18.00, 20.00)	20.00 (19.00, 21.00)	**1.64** (**1.09, 2.53)**	**0**.**019**	1.31 (0.42, 3.70)
Race											
White	106 (61%)	91 (63%)	15 (52%)	1.00 (ref)			95 (60%)	11 (73%)	1.00 (ref)		
Asian	41 (24%)	33 (23%)	8 (28%)	1.47 (0.55, 3.72)	0.4		39 (25%)	2 (13%)	0.44 (0.07, 1.75)	0.3	
Other/multiracial	26 (15%)	20 (14%)	6 (21%)	1.82 (0.59, 5.11)	0.3		24 (15%)	2 (13%)	0.72 (0.11, 2.92)	0.7	
Ethnicity											
Not Hispanic	153 (87%)	130 (89%)	23 (79%)	1.00 (ref)			141 (89%)	12 (75%)	1.00 (ref)	—	
Hispanic	22 (13%)	16 (11%)	6 (21%)	2.12 (0.70, 5.77)	0.2		18 (11%)	4 (25%)	2.61 (0.68, 8.45)	0.13	
School year											
Senior	28 (16%)	21 (14%)	7 (23%)	1.00 (ref)			23 (14%)	5 (29%)	1.00 (ref)		1.00 (ref)
First-year	59 (33%)	51 (34%)	8 (26%)	0.47 (0.15, 1.50)	0.2		58 (36%)	1 (5.9%)	**0.08** (**0.00, 0.53)**	**0**.**024**	0.57 (0.01, 24.7)
Sophomore	38 (21%)	30 (20%)	8 (26%)	0.8 (0.25, 2.60)	0.7		34 (21%)	4 (24%)	0.54 (0.12, 2.25)	0.4	2.52 (0.14, 45.6)
Junior	55 (31%)	47 (32%)	8 (26%)	0.51 (0.16, 1.63)	0.2		48 (29%)	7 (41%)	0.67 (0.19, 2.48)	0.5	3.13 (0.35, 35.3)
Living situation											
With peers/significant other	152 (94%)	128 (96%)	24 (86%)	1.00 (ref)			142 (96%)	10 (71%)	1.00 (ref)		1.00 (ref)
With family	10 (6.2%)	6 (4.5%)	4 (14%)	3.56 (0.86, 13.4)	0.063		6 (4.1%)	4 (29%)	**9.47** (**2.14, 39.1)**	**0**.**002**	**13.00** (**1.98, 95.5)**
Alone	0 (0%)	0 (0%)	0 (0%)				0 (0%)	0 (0%)			
Financial aid	66 (40%)	53 (38%)	13 (46%)	1.39 (0.61, 3.16)	0.4		61 (40%)	5 (33%)	0.74 (0.22, 2.18)	0.6	
Current Relationship status											
In a relationship	79 (44%)	68 (46%)	11 (35%)	1.00 (ref)			72 (44%)	7 (41%)	1.00 (ref)		
Not in a relationship	101 (56%)	81 (54%)	20 (65%)	1.53 (0.69, 3.51)	0.3		91 (56%)	10 (59%)	1.13 (0.41, 3.25)	0.8	
Social group involvement											
None	78 (43%)	62 (42%)	16 (52%)	1.00 (ref)			71 (44%)	7 (41%)	1.00 (ref)		
At least one social group	102 (57%)	87 (58%)	15 (48%)	0.67 (0.30, 1.46)	0.3		92 (56%)	10 (59%)	1.1 (0.40, 3.17)	0.9	
Sports involvement											
None	144 (90%)	120 (90%)	24 (89%)	1.00 (ref)			130 (90%)	14 (93%)	1.00 (ref)		
At least one sports group	16 (10%)	13 (9.8%)	3 (11%)	1.15 (0.25, 3.92)	0.8		15 (10%)	1 (6.7%)	0.62 (0.03, 3.43)	0.7	
Missing	0 (0%)	0 (0%)	0 (0%)				0 (0%)	0 (0%)			
Since 3 months ago, feel…											
Less lonely	107 (60%)	90 (61%)	17 (57%)	1.00 (ref)			96 (59%)	11 (69%)	1.00 (ref)		
Same	39 (22%)	35 (24%)	4 (13%)	0.61 (0.17, 1.77)	0.4		37 (23%)	2 (12%)	0.47 (0.07, 1.87)	0.3	
Lonelier	32 (18%)	23 (16%)	9 (30%)	2.07 (0.79, 5.18)	0.12		29 (18%)	3 (19%)	0.9 (0.19, 3.13)	0.9	
Has a social support network											
Yes	151 (86%)	124 (85%)	27 (90%)	1.00 (ref)			137 (86%)	14 (88%)	1.00 (ref)		
Uncertain	18 (10%)	15 (10%)	3 (10%)	0.92 (0.20, 3.03)	0.9		16 (10%)	2 (12%)	1.22 (0.18, 4.93)	0.8	
No	7 (4.0%)	7 (4.8%)	0 (0%)	0.00 (0.00, 0.00)	>0.9		7 (4.4%)	0 (0%)	0.00 (0.00, 0.00)	>0.9	
Current smoking	13 (7.3%)	9 (6.1%)	4 (13%)	2.27 (0.58, 7.54)	0.2		12 (7.5%)	1 (5.9%)	0.78 (0.04, 4.35)	0.8	
Alcohol consumption											
Never/rare	106 (59%)	87 (59%)	19 (61%)	1.00 (ref)			98 (60%)	8 (47%)	1.00 (ref)		
Moderate/high	73 (41%)	61 (41%)	12 (39%)	0.9 (0.40, 1.97)	0.8		64 (40%)	9 (53%)	1.72 (0.63, 4.81)	0.3	
Drug use											
Never/rare	136 (76%)	114 (77%)	22 (71%)	1.00 (ref)			123 (76%)	13 (76%)	1.00 (ref)		
Monthly/weekly/daily	43 (24%)	34 (23%)	9 (29%)	1.37 (0.55, 3.18)	0.5		39 (24%)	4 (24%)	0.97 (0.26, 2.93)	>0.9	
Recent sexual activity											
No	78 (45%)	67 (46%)	11 (38%)	1.00 (ref)			74 (46%)	4 (27%)	1.00 (ref)		
Yes	97 (55%)	79 (54%)	18 (62%)	1.39 (0.62, 3.23)	0.4		86 (54%)	11 (73%)	2.37 (0.77, 8.82)	0.2	
Missing	0 (0%)	0 (0%)	0 (0%)				0 (0%)	0 (0%)			
Condom use status											
Not sexually active	79 (45%)	68 (47%)	11 (38%)	1.00 (ref)			75 (47%)	4 (25%)	1.00 (ref)		
Sexually active, not using condoms	34 (20%)	30 (21%)	4 (14%)	0.82 (0.21, 2.63)	0.8		31 (20%)	3 (19%)	1.81 (0.34, 8.70)	0.5	
Sexually active, using condoms	61 (35%)	47 (32%)	14 (48%)	1.84 (0.77, 4.49)	0.2		52 (33%)	9 (56%)	3.25 (1.00, 12.5)	0.061	
Response to COVID-19 Symptoms											
Did not seek healthcare or isolate	8 (4.7%)	7 (4.9%)	1 (3.6%)	1.00 (ref)			6 (3.9%)	2 (12%)	1.00 (ref)		
Never had symptoms	137 (81%)	114 (80%)	23 (82%)	1.41 (0.24, 27.1)	0.8		128 (83%)	9 (56%)	0.21 (0.04, 1.58)	0.079	
Isolated	12 (7.1%)	9 (6.3%)	3 (11%)	2.33 (0.24, 53.1)	0.5		8 (5.2%)	4 (25%)	1.5 (0.21, 13.6)	0.7	
Sought healthcare	13 (7.6%)	12 (8.5%)	1 (3.6%)	0.58 (0.02, 16.3)	0.7		12 (7.8%)	1 (6.2%)	0.25 (0.01, 3.12)	0.3	
Effects of COVID-19 on sexual behavior											
No effect	144 (82%)	118 (81%)	26 (84%)	1.00 (ref)			128 (81%)	16 (94%)	1.00 (ref)		
Any effect	31 (18%)	26 (18%)	5 (16%)	0.87 (0.28, 2.33)	0.8		30 (19%)	1 (5.9%)	0.27 (0.01, 1.39)	0.2	
Other	1 (0.6%)	1 (0.7%)	0 (0%)	0.00 (0.00, 0.00)	>0.9		1 (0.6%)	0 (0%)	0.00 (0.00, 0.00)	>0.9	
Current hormonal contraceptive use	80 (45%)	63 (43%)	17 (55%)	1.62 (0.74, 3.57)	0.2		70 (43%)	10 (59%)	1.86 (0.68, 5.35)	0.2	

Median (IQR); *n* (%).

For living situation, we omitted the alone response category from all regressions due to small cell sizes.

Bold values are statistically significant.

For violence perpetration, living with family was associated with increased reports of violence perpetration [aOR = 13.00 (1.98–95.5)].

#### Longitudinal trends in experienced and perpetrated violence: GEE of population average

The GEE sub-sample (*N* = 120) displayed similar outcome trends as the overall sample (Supplementary Table S3). Table 5 shows the unadjusted and adjusted associations estimated by the GEE model. Other/Multiracial identity [aOR = 2.33 (1.25–4.33), ref: White], living with family [aOR = 3.62 (2.24–5.83)], loneliness [aOR = 2.36 (1.36–4.12)], and condom use [aOR = 2.07 (1.20–3.57)] were associated with higher levels of experienced violence over time.

**Table 5:** Unadjusted and adjusted associations of baseline characteristics and experienced and perpetrated violence using generalized estimating equations (GEE).

Baseline characteristic	Overall *N* = 120	Experienced violence			Perpetrated violence		
		Unadjusted		Adjusted *N* = 111	Unadjusted		Adjusted *N* = 113
		OR (95% CI)	*p*-value	aOR (95% CI)	OR (95% CI)	*p*-value	aOR (95% CI)
Age	19.5 (19.0, 20.0)	0.95 (0.80, 1.11)	0.50		1.18 (0.98, 1.43)	0.09	
Race							
White	72 (63%)	1.00 (ref)			1.00 (ref)		
Asian	27 (23%)	1.83 (1.13, 2.95)	**0**.**01**	1.52 (0.87, 2.66)	1.38 (0.77, 2.45)	0.28	
Other/multiracial	16 (14%)	2.07 (1.17, 3.67)	**0**.**01**	**2.33** (**1.25, 4.33**)	1.90 (0.99, 3.65)	0.053	
Ethnicity							
Not Hispanic	103 (88%)	1.00 (ref)			1.00 (ref)		
Hispanic	14 (12%)	0.77 (0.40, 1.45)	0.41	** **	0.98 (0.47, 2.02)	0.95	1.28 (0.82, 2.00)
School year							
Senior	20 (17%)	1.00 (ref)			1.00 (ref)		
First-year	40 (33%)	0.89 (0.50, 1.58)	0.70		0.39 (0.20, 0.74)	**0**.**004**	**0.36** (**0.18, 0.75**)
Sophomore	23 (19%)	1.00 (0.53, 1.88)	0.99		0.61 (0.31, 1.21)	0.16	0.69 (0.32, 1.48)
Junior	37 (31%)	0.65 (0.36, 1.19)	0.16		0.36 (0.19, 0.70)	**0**.**003**	**0.37** (**0.17, 0.81**)
Living situation							
Friends/roommate/significant other	41 (35%)	1.00 (ref)			1.00 (ref)		
Family	76 (65%)	4.10 (2.70, 6.23)	**< 0.001**	**3.62** (**2.24, 5.83**)	3.91 (2.38, 6.43)	**< 0.001**	**4.59** (**2.68, 7.86**)
Financial aid							
No	72 (63%)	1.00 (ref)			1.00 (ref)		
Yes	42 (37%)	1.47 (0.98, 2.21)	0.06		1.68 (1.05, 2.70)	0.03	
Feeling social support							
Yes	93 (78%)	1.00 (ref)			1.00 (ref)		
Don't know	16 (13%)	1.42 (0.77, 2.64)	0.26	** **	0.92 (0.41, 2.06)	0.85	1.18 (0.51, 2.76)
No	11 (9.2%)	1.90 (0.94, 3.84)	0.07	** **	3.23 (1.57, 6.68)	**0**.**001**	**5.30** (**2.19, 12.8**)
Loneliness							
Less lonely	53 (44%)	1.00 (ref)			1.00 (ref)		
Same	26 (22%)	1.08 (0.65, 1.82)	0.76	1.06 (0.59, 1.91)	1.17 (0.66, 2.09)	0.59	
Lonelier	41 (34%)	2.39 (1.51, 3.78)	**< 0.001**	**2.36** (**1.36, 4.12**)	1.19 (0.68, 2.06)	0.54	
Current relationship status							
With relationship	46 (38%)	1.00 (ref)			1.00 (ref)		
Without relationship	74 (62%)	1.27 (0.85, 1.90)	0.25		0.92 (0.58, 1.46)	0.71	
Current smoke							
No	114 (96%)	1.00 (ref)			1.00 (ref)		
Yes	5 (4.2%)	1.58 (0.74, 3.36)	0.24		1.34 (0.56, 3.24)	0.51	
Alcohol consumption							
Never/rare	86 (72%)	1.00 (ref)			1.00 (ref)		
Moderate/high	34 (28%)	0.97 (0.64, 1.47)	0.88		1.14 (0.70, 1.86)	0.59	
Drug use							
Never/rare	102 (85%)	1.00 (ref)			1.00 (ref)		
Monthly/weekly/daily	18 (15%)	0.97 (0.58, 1.62)	0.90		0.82 (0.44, 1.53)	0.53	
Social group involvement							
No social group	43 (36%)	1.00 (ref)			1.00 (ref)		
At least one social group	77 (64%)	0.82 (0.56, 1.22)	0.33		1.20 (0.75, 1.91)	0.45	** **
Sports group involvement							
No sport group	108 (92%)	1.00 (ref)			1.00 (ref)		
At least one sport group	10 (8.5%)	1.02 (0.44, 2.40)	0.96		0.76 (0.25, 2.25)	0.61	
Recent sexual activity							
No	72 (62%)	1.00 (ref)			1.00 (ref)		
Yes	44 (38%)	0.82 (0.55, 1.23)	0.34		1.03 (0.65, 1.66)	0.89	
Condom use status							
Not sexually active	76 (66%)	1.00 (ref)			1.00 (ref)		
Did not use condoms	12 (10%)	0.46 (0.24, 0.89)	**0**.**02**	0.91 (0.42, 1.95)	0.53 (0.24, 1.19)	0.13	
Used condoms	28 (24%)	1.01 (0.65, 1.56)	0.98	**2.07** (**1.20, 3.57)**	1.24 (0.75, 2.06)	0.40	** **
Seeking care							
No care	2 (1.7%)	1.00 (ref)			1.00 (ref)		
No symptoms	103 (86%)	1.32 (0.42, 4.14)	0.63		0.54 (0.18, 1.57)	0.25	
Self-care	6 (5.0%)	2.89 (0.66, 12.57)	0.16		0.51 (0.10, 2.61)	0.64	
Sought care	9 (7.5%)	2.23 (0.57, 8.69)	0.25		0.69 (0.17, 2.73)	0.71	
Sexual behavior affected by COVID-19							
No sex	78 (67%)	1.00 (ref)			1.00 (ref)		
Yes	38 (32%)	0.92 (0.58, 1.46)	0.73		1.07 (0.63, 1.82)	0.79	
Other	1 (0.9%)	1.16 (0.10, 12.88)	0.91		2.24 (0.20, 25.11)	0.51	
Current hormone use							
No	74 (62%)	1.00 (ref)			1.00 (ref)		
Yes	46 (38%)	0.87 (0.58, 1.29)	0.47		1.02 (0.64, 1.61)	0.95	

Bold values are statistically significant.

Living with family [aOR = 4.59 (2.68–7.86)] and no social support [aOR = 5.30 (2.19–12.80)] were associated with higher levels of perpetrated violence over time, while school year (First year: aOR = 0.36 [0.18–0.75], Junior: aOR = 0.37 [0.17–0.81], ref: Senior) was associated with lower levels of perpetrated violence.

## Discussion

In this sample of college women, self-reported experience of violence was high, and the majority of reported violence was verbal. About one-half of respondents experienced verbal/physical violence, and two-fifths of respondents perpetrated verbal/physical violence. Aside from a slight increase at T3, these outcomes decreased over time. Living with family compared with peers/significant others consistently increased odds of experienced (T1-T3) and perpetrated (T1-T4) violence. Low perceived social support also increased odds of violence experienced (T1-T3) and perpetrated (T1, T3) at most time points. Other/Multiracial identity was associated with higher odds of violence experience at T3. We observed proportions up to four times larger than levels estimated from a national survey of college students conducted during the same time. In the national survey, approximately 12% of female respondents experienced verbal violence from partners and non-partners (16–18, 37, 38).

The geographic location of our sample might contribute to this difference. NYC and its metro area were largely considered the epicenter of the early COVID-19 epidemic in America, characterized by the country's highest population density and early case detection and mortality rate. As a result, intensified pandemic-related stress and prolonged shelter-in-place directives may have influenced verbal violence perpetration and experience, respectively.

Data from a national survey of college students found verbal violence experience from all sources was relatively stable during the early pandemic and comparable to pre-pandemic levels (15–18). Conversely, among our study population, violence decreased over the study period, alongside relaxing of shelter-in-place and social distancing protocols, returning to in-person learning, and expanding eligibility and deployment of COVID-19 vaccines. This downward trend likely also reflects increased availability of access by violence victims/perpetrators to the suite of comprehensive support services offered by the host college, spanning the full-spectrum of medical care, psychosocial health care, and wrap-around coordination services. Our survey did not measure uptake of these services; thus, future examinations should consider exploring the potential effect of institutional services and resources on violence outcomes. The slight increase in violence at T3 corresponded with a mild uptick in NYC COVID-19 cases and summer vacation, possibly coinciding with changes in living situation, academic structure, and supportive social outlets, such as sports or arts groups. Of note, though we detected a downtrend trend in violence over the study period, even at the final timepoint, we still observed violence experiences that were five percentage points higher than national estimates (18).

The preponderance of experienced and perpetrated violence was verbal. Although not always viewed or perceived as abuse (39, 40), verbal violence can negatively affect health, psychosocial wellbeing, and development across the life course. A recent systematic review showed that college students' experience of verbal abuse can lead to emotional problems and coping issues, depression and poor mental wellbeing, increased alcohol use, and neurological vulnerabilities. Importantly, these experiences can outwardly manifest as cognitive desensitization, maladaptive beliefs regarding conflict, and increased perpetration and victimization of abuse and violence (41). Verbal violence from partners, peers, and/or adults can negatively affect academic achievement, self-esteem, reproductive decision-making, and sociability (42–45); it can also precede and/or co-occur with physical or sexual violence (42, 46, 47).

Although not commonly reported, we observed that physical violence among experiencers and perpetrators nearly doubled and tripled, respectively, between T1 and T3. A prior meta-analysis found connections between an increased propensity of woman-perpetrated physical aggression among those reporting interpersonal traumatic events and symptoms of post-traumatic stress disorder (PTSD) (48). It is possible that pandemic-induced PTSD symptoms manifested in our participants as time progressed and, subsequently, contributed to the perpetration of physical violence (49). The linear, concurrent increase in physical violence experience and perpetration between T1 and T3 also suggests some form of bidirectionality between perpetration and experienced. Also, unlike verbal violence, which consistently had lower perpetration than experience, physical violence perpetration exceeded experience after T2. These observations are interesting because pre-pandemic research among women survivors of IPV has shown that violence experience begets perpetration; independent perpetration (i.e., not in response to experiencing violence) was extremely rare (50). The dynamics of violence perpetration by women outside of intimate partner settings may differ; given the paucity of research, additional investigation of woman-perpetrated violence is needed to understand this phenomenon better.

We hypothesized that social connectivity would be associated with violence perpetration and experience, and we found living situation was consistently associated with violence; living with peers/significant others compared to living with family was associated with reduced violence experiences (T1-T3, GEE) and perpetration (T1-T4, GEE). Interruptions in in-person learning and varying availability of on-campus housing because of isolation requirements likely affected decision-making around choice of residence during successive waves of the pandemic. Higher financial, food and health insecurity as a result of the pandemic and associated lockdown policies may have contributed to increased household stress, which in turn may have impacted the likelihood of experiencing or perpetrating violence (51, 52). It is unclear from our findings whether violence was perpetrated by/enacted on family members themselves, whether there were non-familial members of the household engaged in violence, or whether the residential dynamics led students to have extra-residential relationships that were more likely to contain violence (although the latter is less likely give that relationship status on its own was not linked with violence). Regardless, our findings highlight that students living with family are particularly vulnerable to violence and merit intensified in-person and/or remote outreach and support services for harm mitigation. Flexible housing services, such as enabling early return to campus and/or staying on campus over breaks, may also help decrease violence experience and/or perpetration.

In our study, perceived lack of social support was strongly associated with experience and perpetration of violence at selected timepoints and in the GEE model. Our findings complement previous research, which has found that greater social support and housing services were associated with lower experience of abuse among domestic violence survivors during the pandemic (53). Social support may help reduce violence experiences by offering individuals a safe space to retreat to when disagreements are escalating; relatedly, supportive peers may help deescalate and/or regulate emotions before they manifest as enacted verbal/physical violence. Social support interventions have had notable success in increasing social networks and minimizing negative mental health outcomes among violence survivors (54). Given the modifiability of social support, colleges should make increased efforts to foster community and create social cohesion, which can attenuate violence among student populations (45).

At T3 and in the GEE model, racial identity was linked with violence; students identifying as Other/Multiracial had significantly higher violence experience than White-identifying students. Before the pandemic, non-White race has been identified as a variable associated with an increased risk of IPV (39). Other authors have highlighted the unequal experience of violence by women from racial and ethnic minority backgrounds during the pandemic (55–57). The social and economic ramifications of pandemic disruptions had an outsized impact on people who already faced marginalization (55–57). Notably, T3 was the first data collection period that did not coincide with the disbursement of COVID-19 stimulus checks, as the second and final checks were distributed at the beginning of T1 and T2 (58). Excluding New York (59), several states also ended pandemic employment benefits in June 2021 (60). Since financially marginalized populations, including non-dependent college students, benefitted considerably from this governmental support (61), financial strain and worry caused by the lack of checks during this survey period might have created a household milieu that facilitated verbal and/or physical violence (62, 63). Given the student body makeup of the host college, there is also a possibility that some of these students were international students, who were unable to travel home due to travel restrictions (64, 65). Since most domestic students likely left campus/NYC at T3 for summer break, isolation from peers, friends, and family abroad could have raised tensions among international students. Given their unique circumstances, greater insight into the experiences of international students during COVID-19 is needed.

In our study, moderate/high alcohol consumption was not associated with violence. Alcohol consumption and substance use were noticeably low among our cohort, which could suggest either that social desirability bias affected reported responses or that the study population differed in behavior compared with other college-aged populations (66). This finding may also suggest that the high levels of violence experience and perpetration observed in our sample are not meaningfully attributable to alcohol use, either as a coping mechanism for experience or an antecedent for perpetration. However, predictive longitudinal analyses would better illuminate the temporality of these relationships and assess if our findings are null due to contemporaneous measurements.

At T3, students who were sexually active and not using condoms had lower violence experience compared to those who were not sexually active. It is possible that here, lack of condom use signified trust and stability in relationships. Findings from the GEE models support this notion, as condom use—potentially indicating relational instability—and violence experience were positively associated.

There are several limitations to our study. First, since the violence questions were framed around partners or people living in the same domicile, we cannot discern the type of individuals involved in these violence experiences/perpetrations. Future research should try to distinguish between the different types of violence (e.g., intimate partner violence, familial violence, etc.) Second, the last follow-up survey period was in November-December 2021, and we could not assess the correlates of violence experience and perpetration as pandemic experiences slowly became normalized. Third, the analysis relied on self-reported data, which might have introduced recall or social desirability bias. Fourth, since we enrolled participants at a NYC college, generalizability may be limited. Fifth, we cannot establish temporality between the outcomes and correlates given our cross-sectional analyses; however, the longitudinal design enables us to identify temporally persistent and/or unique associations. Sixth, we did not measure exposure to or uptake of specific college-provided services that may have influenced violence prevention, post-care, or perpetration, suggesting there could be some level of unmeasured confounding affecting our analyses. Seventh, we analyzed physical and verbal violence in combination, and we did not measure sexual violence; future research should explore these individually using a larger sample size. Finally, small cell sizes for select variables may have introduced bias and/or affected model convergence.

## Conclusions

Violence against women is a persistent global public health crisis that worsened during COVID-19, owing to pandemic countermeasures and increases in stressors worldwide. Historically, college women have been particularly vulnerable to violence, warranting investigation of their experiences during COVID-19. In our sample, violence experience was remarkably high, with verbal violence representing the majority of violence experienced and perpetrated. Living situation and level of social support emerged as important correlates. Understanding modifiable correlates of violence can guide the delivery of interventions to key populations to help mitigate social, relational, and behavioral factors that may increase vulnerability to violence in current and future pandemics. As part of pandemic health preparedness, universities should strengthen violence prevention and support systems for young women by developing programming to promote social cohesion; universities should then assess the impact of their programming on reports of violence in their community. In addition to impacting university practices in this manner, our findings can be used to promote development of university policy on violence and to guide directions for future violence research.

## Data Availability

The datasets presented in this article are not readily available because of their sensitive nature. Requests to access the datasets should be directed to Deborah A. Theodore, dat2132@cumc.columbia.edu.
