# Supplementary material for: Correlates of verbal and physical violence experienced and perpetrated among cisgender college women: serial cross-sections during one year of the COVID-19 pandemic
Source: Front Reprod Health. 2024 Jul 25;6:1366262. doi: 10.3389/frph.2024.1366262 (PMC11306199; doi:10.3389/frph.2024.1366262)
Supplement: Supplementary file 2 [file Table2.docx]

**Supplemental Material 2. Correlate definitions**

| **Correlate** | **Collection frequency** | **Definition** |
| --- | --- | --- |
| **Demographics** |  |  |
| Age | T1 only | Continuous age in years |
| Race | T1 only | White, Asian, or Other/Multiracial  Other/Multiracial includes multiple responses and responses of American Indian/Alaskan Native and Native Hawaiian/Pacific Islander, Black/African American (low representation), and Other |
| Ethnicity | T1 only | Hispanic ethnicity vs. Not |
| School year | T1 only | First-year, Sophomore, Junior, or Senior |
| Living situation | T1-T4 | Lived with Family, Peers/Significant Others, or Alone |
| Financial aid status | T1-T4 | Received need-based financial aid vs. not |
| **Social variables** |  |  |
| Relationship status | T1-T4 | In a relationship (Partnered/Married/Engaged/Multiple relationships) vs. Single. |
| Social group involvement | T1-T4 | Involved in at least one social group (Peer educator/advisor, Resident assistant, Student government, Dance/theater group, Musical group, Teaching assistant, Other) vs. None |
| Sports group involvement | T1-T4 | Involved in at least one sports group (Club sports, Intramurals, Varsity) vs. None |
| Loneliness | T1-T4 | Felt less lonely, same amount of lonely, or lonelier than 3 months ago |
| Social support | T1-T4 | Respondents were asked if they had a social support network where they lived, with response categories including Yes, Uncertain, or No |
| **Substance use** |  |  |
| Smoking/vaping | T1-T4 | Currently smoked cigarettes or vapes vs. Not |
| Alcohol use | T1-T4 | Categories were created based on drinking frequency over the last 30 days, encompassing Never, Rare (<1-2 days per week), Moderate (1-2 days per week), and High (3-4 days per week or more frequent). Moderate and High categories were combined because the High category had small proportions. Never and Rare categories were combined to reflect the infrequency of the behavior. |
| Drug use | T1-T4 | Categories were created based on frequency of drug use over the last 30 days, including Never, Rare (less than once a month), Moderate (1-3 days a month), and High (1-2 days a week or more frequent). Moderate and High categories were combined because the High category had small proportions. Never and Rare categories were combined to reflect the infrequency of the behavior. |
| **Sexual behaviors** |  |  |
| Recent sexual activity | T1-T4 | Sexually active in the last 3 months vs. Not |
| Condom use | T1-T4 | Used condoms vs. Did not use condoms vs. Not sexually active |
| Change in sexual behavior due to COVID-19 | T1-T4 | COVID-19 related social distancing affected their sexual behavior vs. Not vs. Other |
| **Service utilization** |  |  |
| Care-seeking behaviors based on COVID-19 symptoms | T1-T4 | Sought healthcare (Telemedicine, Non-urgent care visit, Urgent care visit, Emergency room, Hospital admission, ICU admission) vs. Never had symptoms vs. Isolated vs. Did not seek healthcare / isolate |
| Current hormonal contraceptive use | T1-T4 | Using hormonal contraceptives (oral contraceptives, implant, IUD, vaginal ring, injectables, patch) vs. None |

All correlates were created using self-reported data
