# Supplementary material for: Correlates of verbal and physical violence experienced and perpetrated among cisgender college women: serial cross-sections during one year of the COVID-19 pandemic
Source: Front Reprod Health. 2024 Jul 25;6:1366262. doi: 10.3389/frph.2024.1366262 (PMC11306199; doi:10.3389/frph.2024.1366262)
Supplement: Supplementary file 3 [file Table3.docx]

| **A.**  **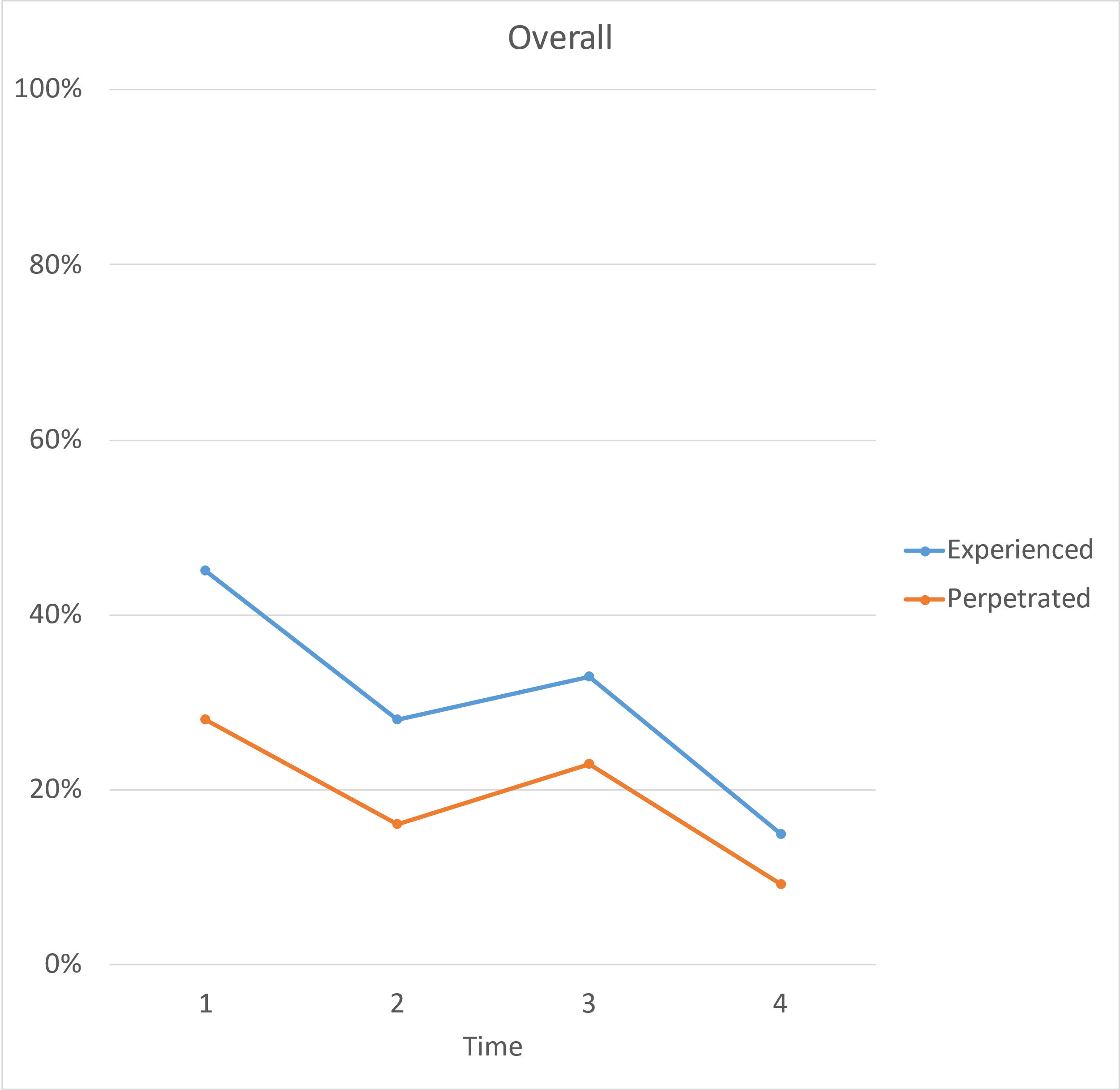** | **B.**  **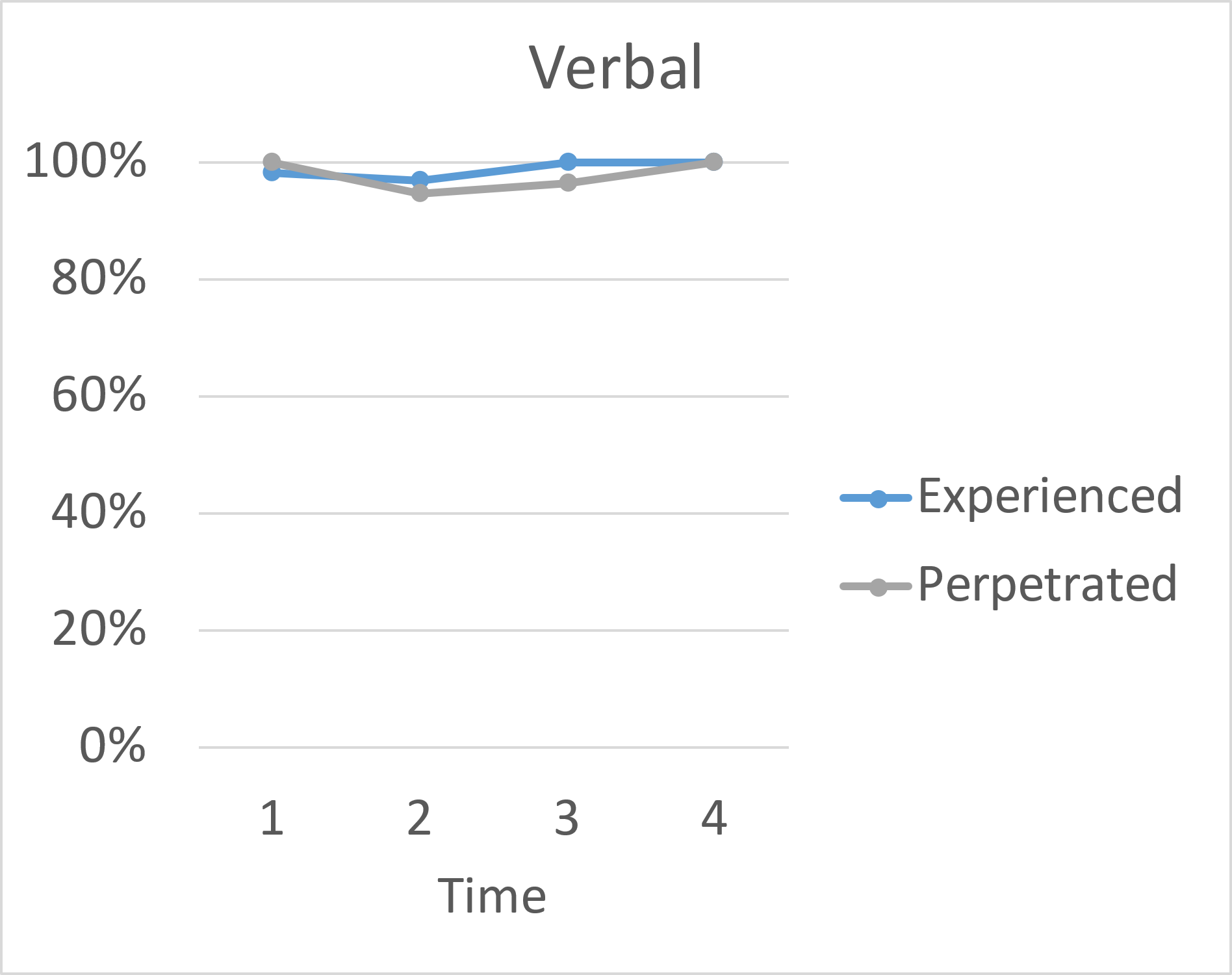** |
| --- | --- |
|  | **C.**  **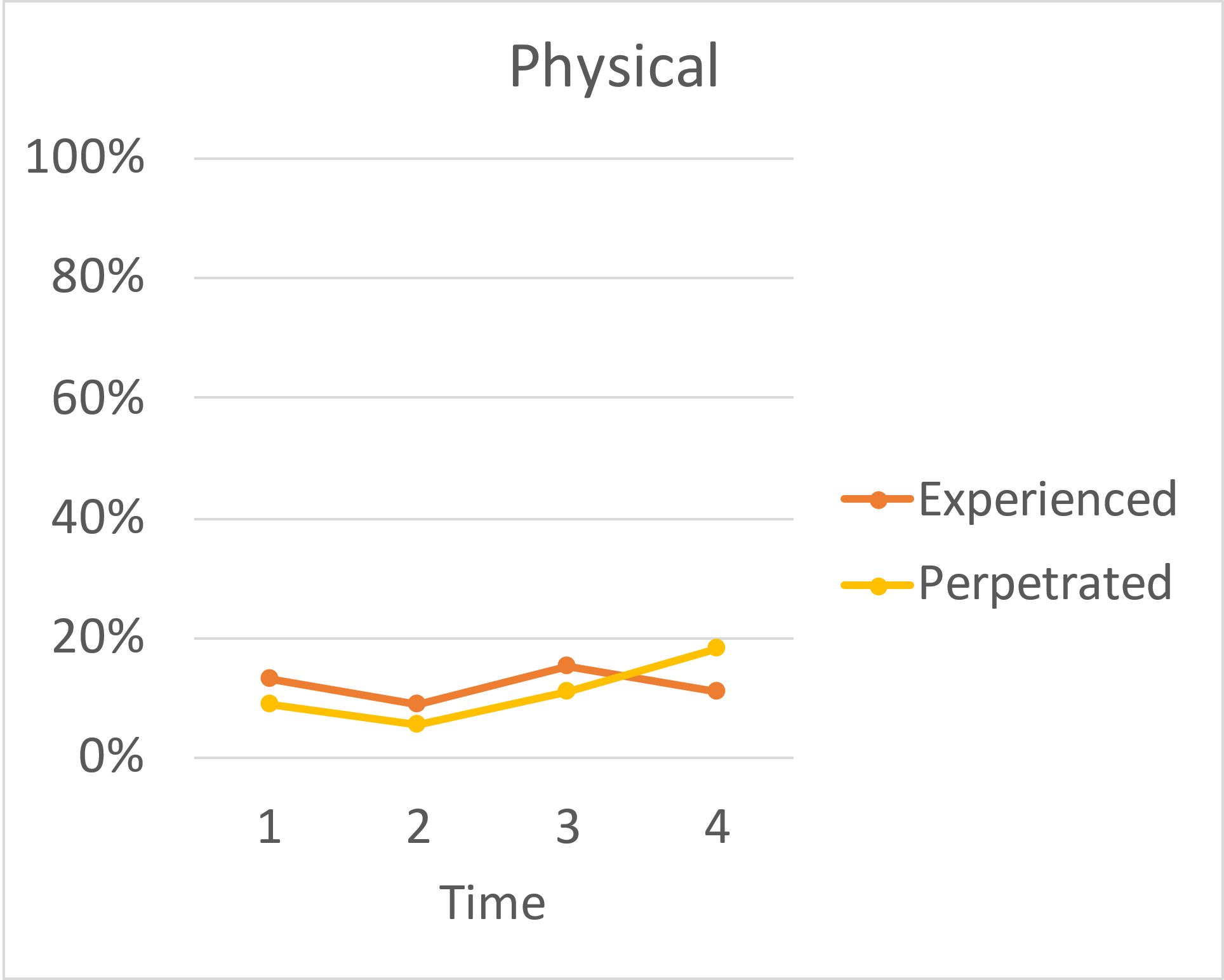** |

**Supplemental Material 3. Experience and perpetration of violence over time among those with outcome data across all timepoints (N=120).** Panel A shows the joint experience of verbal and physical violence (blue) and the joint perpetration of verbal and physical violence (orange). Panel B shows the experience (blue) and perpetration (gray) of verbal violence. Panel C shows the experience (orange) and perpetration (yellow) of physical violence.
